# Evaluation of Amoebicidal Potential of Paneth Cell Cryptdin-2 against *Entamoeba histolytica*

**DOI:** 10.1371/journal.pntd.0001386

**Published:** 2011-12-20

**Authors:** Simran Preet, Sanjay Bharati, Geeta Shukla, Ashwani Koul, Praveen Rishi

**Affiliations:** 1 Department of Microbiology, Basic Medical Sciences Block, Panjab University, Chandigarh, India; 2 Department of Biophysics, Basic Medical Sciences Block, Panjab University, Chandigarh, India; Christian Medical College, India

## Abstract

**Background:**

Amoebiasis is a major public health problem in tropical and subtropical countries. Currently, metronidazole is the gold choice medication for the treatment of this disease. However, reports have indicated towards the possibility of development of metronidazole-resistance in *Entamoeba* strains in near future. In view of the emergence of this possibility, in addition to the associated side effects and mutagenic ability of the currently available anti-amoebic drugs, there is a need to explore newer therapeutics against this disease. In this context, the present study evaluated the amoebicidal potential of cryptdin-2 against *E. histolytica*.

**Methods/Principal Findings:**

In the present study, cryptdin-2 exhibited potent *in-vitro* amoebicidal activity against *E. histolytica* in a concentration dependent manner at a minimum amoebicidal concentration (MAC) of 4 mg/L. Scanning electron microscopy as well as phase contrast microscopic investigations of cryptdin-2 treated trophozoites revealed that the peptide was able to induce significant morphological alterations in terms of membrane wrinkling, leakage of the cytoplasmic contents and damaged plasma membrane suggesting a possible membrane dependent amoebicidal activity. N-phenyl napthylamine (NPN) uptake assay in presence of sulethal, lethal as well as twice the lethal concentrations further confirmed the membrane-dependent mode of action of cryptdin-2 and suggested that the peptide could permeabilize the plasma membrane of *E. histolytica*. It was also found that cryptdin-2 interfered with DNA, RNA as well as protein synthesis of *E. histolytica* exerting the highest effect against DNA synthesis. Thus, the macromolecular synthesis studies correlated well with the observations of membrane permeabilization studies.

**Significance/Conclusions:**

The amoebicidal efficacy of cryptdin-2 suggests that it may be exploited as a promising option to combat amoebiasis or, at least, may act as an adjunct to metronidazole and/or other available anti-amoebic drugs.

## Author Summary

Intestinal amoebiasis, caused by *Enatmoeba histolytica* continues to be a major public health problem in tropical and subtropical countries and is considered to be the third principal parasitic disease responsible for mortality in the world. In addition to the mutagenic ability and known toxicity of conventional anti-amoebic drugs, there are reports indicating the emergence of treatment failures to these drugs. Therefore, there has been a considerable interest in exploring the potential of various antimicrobial peptides having higher efficacy and lower toxicity to combat such parasitic infections. Herein, we present the amoebicidal efficacy of cryptdin-2, a Paneth cell alpha-defensin against *E. histolytica*. Cryptdin-2 was found to decrease the number of trophozoites of *E. histolytica* in a concentration dependent manner. By and large, cryptdin-2 could retain its amoebicidal activity in the presence of cations, bile salts and at various pH values. Microscopic analysis and N-phenyl napthylamine (NPN) uptake assay revealed membrane dependent amoebicidal action of the peptide. It was also demonstrated that cryptdin-2 has the potential to target the intracellular macromolecular synthesis machinery of *Entamoeba*. Based on these results, cryptdin-2 seems to be a promising agent for the development of novel therapeutics against amoebiasis or at least may act as an adjunct to conventional antibiotics against *E. histolytica*.

## Introduction

Amoebiasis is a major public health problem in tropical and subtropical countries and is considered to be the third leading cause of death amongst parasitic diseases worldwide [1]. The incidence of this disease has currently been estimated to be approximately 50 million people with symptomatic infections while causing 100,000 deaths annually, essentially in developing countries [2]–[4]. Amoebiasis, is manifested by the transmission of cysts of *Entamoeba histolytica* through the fecal-oral route from contaminated water or food. Trophozoites of this primitive parasite are able to invade the intestinal mucosa causing dysentery, fever and abdominal pain. These trophozoites often spread to other organs such as liver thereby causing liver abscesses and death in severe cases [5].

Metronidazole is the most widely used medication to combat luminal and hepatic amoebiasis, but it is toxic and might be mutagenic for patients when used at high doses or as long term treatment [6]. It is usually well tolerated but may cause nausea, vomiting and abdominal cramps in addition to its metallic taste [7], [8]. Although drug-resistant amoebae are not as frequently described as are drug-resistant malaria parasites, differences in drug susceptibilities among strains of amoebae have been reported [9], [10]. Reports on treatment failure also indicate that drug resistance may become clinically important in the near future [11]. It provides impetus to the efforts to identify and exploit alternative anti-amoebic therapies.

A multitude of preliminary studies suggest that cationic antimicrobial peptides (AMPs) represent a promising route towards developing new, efficient antiparasitic therapies [12]–[14]. Among naturally occurring AMPs, defensins form a unique family of cysteine-rich cationic polypeptides with 3–4 disulfide bridges [15]. Mouse enteric alpha-defensins, present in Paneth cell apical granules are called cryptdins (for crypt defensins). Human Paneth cells code for two α-defensins (HD-5 and HD-6) while six alpha-defensins (cryptdins 1–6) have been characterized from murine small intestine [16]. Amongst these cryptdin isoforms, cryptdin-1 and cryptdin-2 are the most abundant peptides [17]. Due to the additional pore forming property possessed by cryptdin-2, this peptide was employed in the present study [18]. Recently, we have demonstrated that cryptdin-2 possesses a strong *in-vivo* therapeutic potential against murine salmonellosis without exhibiting any toxicity as indicated by liver and kidney function tests [19]. Additionally, it was found to exhibit very low cytotoxicity towards macrophages even at a concentration twice that of the MBC [19].

The giardicidal effect of cryptdins has been investigated earlier [20] in addition to their bactericidal [21], [22] and anti-viral properties [23]. However, the paucity of information regarding the activity of cryptdins against *Entamoeba histolytica* is surprising in view of the fact that the protozoan comes in direct contact with these peptides in the intestinal lumen (where cryptdins are secreted) during penetration through the mucus layer and entry into the crypts. Therefore, the present study was designed to assess the amoebicidal potential of Paneth cell cryptdin-2 against *Entamoeba histolytica*.

## Materials and Methods

### Parasite and culture conditions

Standard strain of *E. histolytica* (HM1: IMSS) initially procured from Dr. Alok Bhattacharya, Professor, Jawaharlal Nehru University, New Delhi, India and being maintained in the Department of Parasitology, Post Graduate Institute of Medical Education and Research, PGIMER, Chandigarh, India was used in the present study. Trophozoites were maintained axenically in trypticase-yeast extract iron-serum (TYI-S-33) medium in screw-capped tubes. The media contained tryptone: 2 g, yeast extract: 1 g, glucose: 1 g, NaCl: 200 mg, K_2_HPO_4_: 100 mg, KH_2_PO_4_: 60 mg, L-cysteine-HCl: 100 mg, L-ascorbic acid: 20 mg, ammonium citrate: 2.28 mg and 75 ml of distilled water. pH was adjusted to 6.8–7.0±0.2 using 1N NaOH. Antibiotic mixture (streptomycin: 0.5 ml, penicillin 0.5 ml and zentamycin 0.2 ml),10% inactivated horse serum and 3% vitamin mixture were also added to the medium. Serum was inactivated by keeping it at 56°C for 30 minutes. *E. histolytica* cultures in log phase were used for *in vitro* inhibition assay. Prior to isolation, dead parasites were removed by aspiration. Live trophozoites were detached by chilling on ice for 10 min, harvested by centrifugation (300 g, 20 min), and re-suspended at a concentration of 2×10^5^ trophozoites/ml in 5 mM HEPES (N-2-hydroxyethylpiperazine- N′-2-ethanesulfonic acid) (pH 7.5).

### Metronidazole and synthetic cryptdin-2

Metronidazole was procured as a pure salt from Sigma-Aldrich Co., St. Louis, MO., USA. The stock solution (100 mg/L) of the drug was prepared in dimethyl sulphoxide (DMSO) and stored at −20°C till use. Chemically synthesized peptide with an amino acid sequence LRDLVCYCRTRGCKRRERMNGTCRKGHLMYTLCCR, identical to the sequence of mouse Paneth cell cryptdin-2 with disulphide linkages between Cys^I^-Cys^VI^, Cys^II^-Cys^IV^, Cys^III^-Cys^V^, was obtained from Taurus Scientific, USA. It was suspended in 0.01% acetic acid, stored as a stock solution of 100 mg/L at −20°C and was used within 3 weeks.

### *In-vitro* susceptibility of *Entamoeba histolytica*

*In vitro* susceptibility of *E. histolytica* to cryptdin-2 and metronidazole was determined by the method as described by Cedillo-Rivera and Munioz [24]. Briefly, 5×10^5^ trophozoites/ml of *E. histolytica* were incubated with different concentrations (0.5–64 mg/L) of cryptdin-2 and metronidazole in TYI-S-33 medium at 37° C for 48 h. Control cultures contained the same volume of 0.01% acetic acid. At the end of the treatment period, trophozoites were counted using a haemocytometer by trypan blue dye exclusion method and the minimum amoebicidal concentration (MAC) (at which there was 99.99% inhibition of growth) was calculated by monitoring the number of trophozoites at various concentrations with respect to the control after 48 hours of incubation.

### Effect of ionic strength on amoebicidal activity of cryptdin-2

This was done by the similar method as described above with a slight modification.Various concentrations of NaCl and/or KCl (i.e 10, 50, 100 and 200 mM) were added to TYIS-33 medium in order to evaluate the effect of monovalent cations on the amoebicidal activity of cryptdin-2. Similarly, the divalent cations, CaCl2 and/or MgCl2 were added at various concentrations (1, 2, 5, 10, and 20 mM) to TYIS-33 the medium and MAC was calculated after 48 h of incubation.

### Effect of pH and bile salts

The effect of pH and bile salts on the amoebicidal activity of the cryptdin-2 was tested by determining its MACs in the presence of bile salts and at various pH values by the method as described above with a slight modification. The pH of the assay medium was altered by adding either 5 M HCl or NaOH. The amoebicidal activity was tested at pH values ranging from pH 5 to pH 8. Similarly, for evaluating the effect of bile salts, TYI-S-33 medium used in the above assay was supplemented with 0.3% of sodium taurocholate and sodium deoxycholate and MAC was calculated.

### Morphological alterations induced by cryptdin-2 in *E. histolytica*

To assess the effect of cryptdin-2 on the morphology of *Entamoeba histolytica*, 3×10^3^ trophozoites/ml were incubated with 2 mg/L of cryptdin-2 (sub-lethal concentration) for 60 min at 37°C and effect on morphology of the amoebae was examined by simple light microscope (400×) as well as phase contrast microscope (600×). Trophozoites incubated with 0.01% acetic acid served as controls. The ultrastuctural changes induced by cryptdin-2 were studied by scanning electron microscopy (SEM). For the SEM study, trophozoites were fixed in 2% glutaraldehyde (1 h at room temperature), postfixed in 2% osmium tetroxide (30 min in the dark), dehydrated in a series of graded alcohol baths, and then subjected to critical-point drying in CO2. Finally the samples were mounted on aluminium stubs, coated with gold-palladium at a thickness of 200A°, and examined for the change in morphology by scanning electron microscope (JEOL JEM 1600 model).

### Membrane permeabilization assay

The ability of cryptdin-2 to permeabilize the membrane of *E. histolytica* was investigated using N-phenyl napthylamine (NPN) uptake assay [25]. To evaluate the effect at different peptide to lipid ratios, sub-inhibitory as well as higher concentrations of cryptdin-2 were used. Briefly, 20 µl of mid-log phase trophozoites of *E. histolytica* (1×10^6^ trophozoites/ml) were suspended in 100 µl of 5 mM HEPES (pH 7.4) containing 10 µM NPN in 1.5 ml tubes. After 5 min of incubation, cryptdin-2 (0.5 MAC, MAC and 2MAC) was added, and the increase in fluorescence of NPN was monitored at an excitation and emission wavelength of 340 nm and 415 nm respectively, with slit widths of 5 nm. 10 µM EDTA (a known membrane permeabilizer) was added to the control tubes. The emission and excitation wavelength were determined after analyzing the fluorescence spectrum of NPN in presence of *Enatamoeba histolytica* trophozoites (without any membrane permeabilizer) at different excitation wavelengths using a LS55- Perkin-Elmer luminescence spectrophotometer. Relative fluorescence units (fluorescence value of cell suspension with the test substance and NPN subtracted with the corresponding value of the cell suspension and NPN without the test substance) were measured at different time intervals.

### Effect on macromolecular synthesis (pulse labeling studies)

The effect of cryptdin-2 on the incorporation of [^3^H] thymidine, [^3^H]- uridine, and [^3^H] leucine (Board of Radiation and Isotope Technology (BRIT, India) in amoebic DNA, RNA, and proteins respectively, was also studied. In brief, mid-log phase cultures with1×10^6^ trophozoites/ml were incubated with 0.5MAC, MAC and 2× MAC of cryptdin-2 in presence of 2.5 µl/ml of either [methyl-5-^3^H] thymidine (18000 mCi/mmol), [5-^3^H] uridine (16000 mCi/mmol), or C14-[L-leucine (210 mCi/mmol) for different time points. After the incubation, trophozoite suspensions were added to ice-cold 10% trichloroacetic acid, mixed well, and allowed to stand on ice for 40 min. Samples were then collected onto nitrocellulose filters. The filters were washed thoroughly with 5% trichloroacetic acid and 70% ethanol, dried , placed in 7 ml scintillation cocktail (Sigma Aldrich Chemicals, St. Louis, MO, USA) and the bound radioactivity was then counted in liquid scintillation counter for 1 min for each filter. (Counts per minute, cpm). The radioactivity incorporated in the trophozoites was calculated using a standard curve plotted between cpm and radioactivity (mCi) for all the three radiolabelled precursors (at various concentrations. The calculated radioactivity was then converted to molar concentrations of each of the precursor by using the following formula:

Moles of precursor incorporated = Calculated radioactivity/specific activity (for each precursor)

### Statistical Analysis

Data were expressed as mean ± standard deviation of three to five independent experiments. Statistical analysis was done by Student's unpaired *t* test and one way analysis of variance (ANOVA) followed by pair wise comparison procedures (Tukey test) using Jandel Sigma Stat Statistical Software, version 2.0. In all cases, statistical significance was defined as p≤0.05.

## Results

### Amoebicidal activity of cryptdin-2

**Figure 1:**
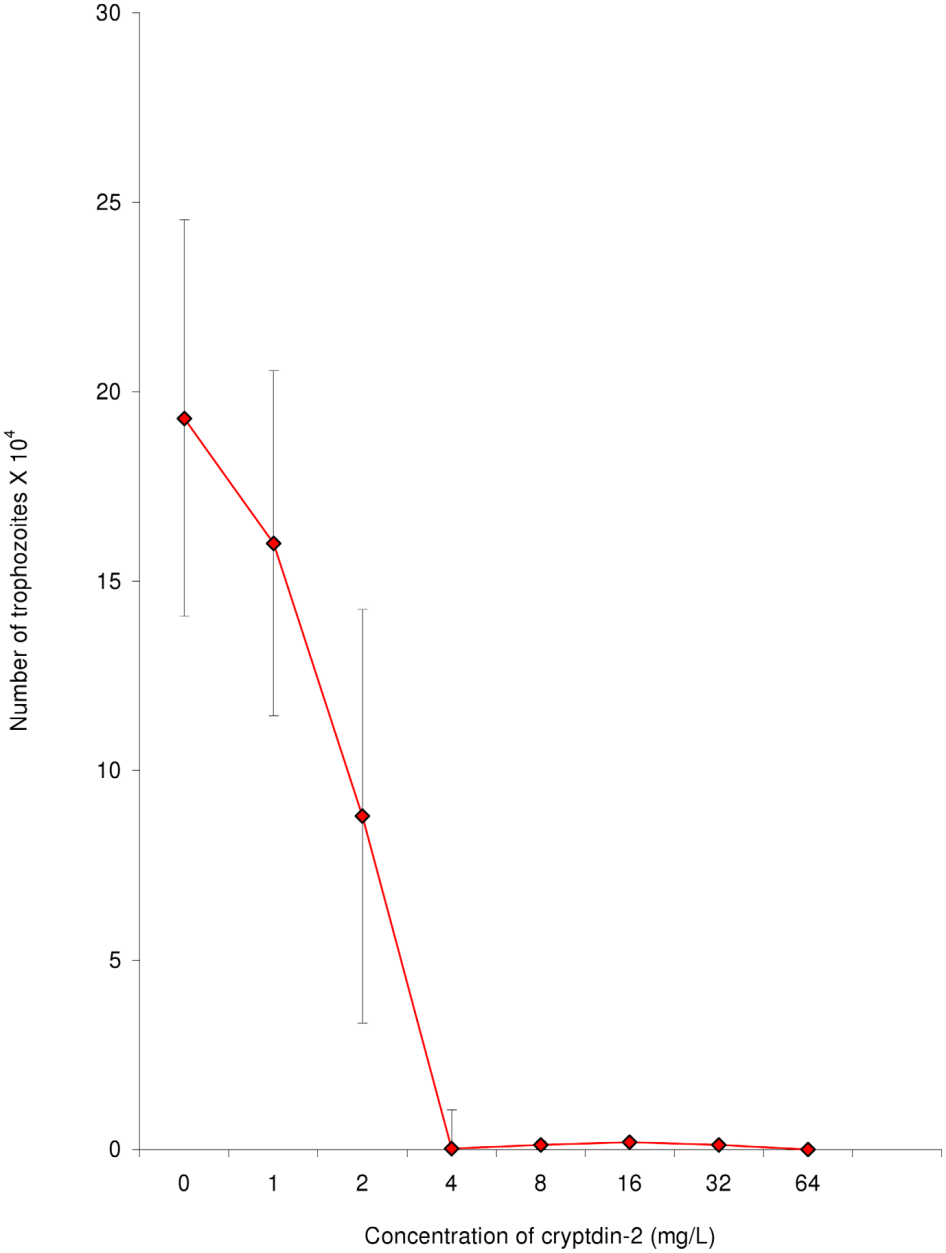
Minimum amoebicidal concentration of cryptdin-2. Decrease in trophozoite count of *E. histolytica* in the presence of various concentrations of cryptdin-2. Values are expressed as mean ± SD of five independent experiments.*p<0.001 vs. number of trophozoites in control (after 24 hours).

Cryptdin-2 and metronidazole inhibited the growth of *E. histolytica* trophozoites in a concentration dependent manner while an increase in trophozoite count was observed in control as compared to the initial count after 48 hours. Minimum amoebicidal concentrations of cryptdin-2 and metronidazole were evaluated to be 4 mg/L and 4.5 mg/L respectively as more than 99.9% decrease (p<0.001) in trophozoite counts at this concentration was observed as compared to the control (Fig. 1).

### Effect of ionic strength on amoebicidal activity

**Figure 2:**
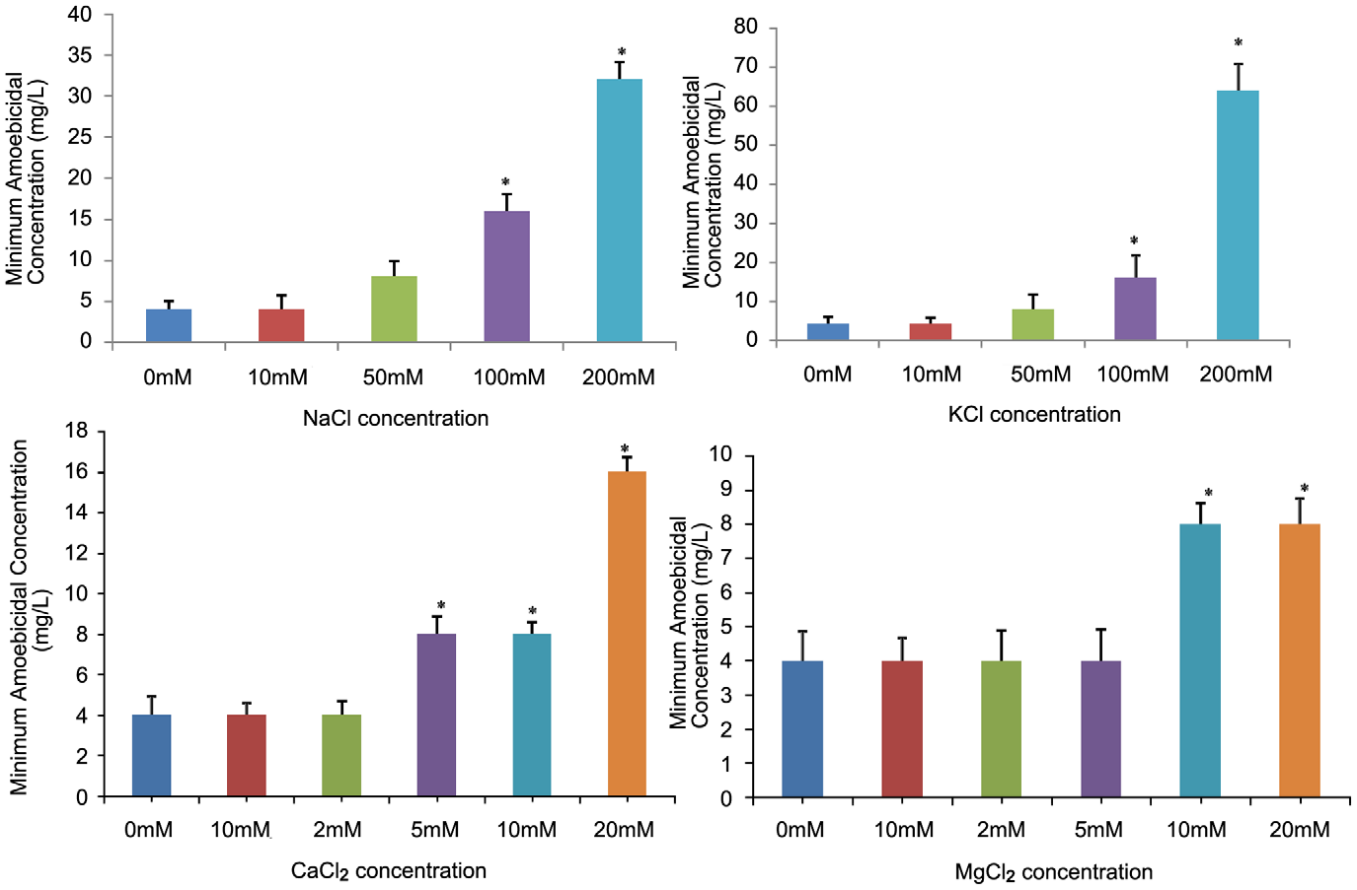
Effect of ionic strength on amoebicidal activity of cryptdin-2. A) Minimum amoebicidal concentrations (MACs) of cryptdin-2 in presence of various concentrations of NaCl (mM) *p<0.05 vs. MAC in absence of NaCl. B) Minimum amoebicidal concentrations (MACs) of cryptdin-2 in presence of various concentrations of KCl (mM) *p<0.05 vs. MAC in absence of KCl. C) Minimum amoebicidal concentrations (MACs) of cryptdin-2 in presence of various concentrations of CaCl_2_ (mM) *p<0.05 vs. MAC in absence of CaCl_2_. D) Minimum amoebicidal concentrations (MACs) of cryptdin-2 in presence of various concentrations of MgCl_2_ (mM) *p<0.05 vs. MAC in absence of MgCl_2._Values are Mean ± SD of five independent experiments.

The MAC was not found to be influenced in the presence of 10 mM NaCl. However, the values increased to 8 mg/L, 16 mg/L (p<0.05), 32 mg/L (p<0.05) at 50, 100 and 200 mM NaCl concentrations (Fig. 2A). Similarly, no antagonistic effect of 10 mM KCl (a concentration much higher than its approximate plasma physiological concentrations) on the MAC values was observed [26], though the MAC values increased at higher concentrations of KCl (Fig. 2B). Overall, the results exhibited that although the MAC values were increased at higher concentrations of both the monovalent cations, complete loss of activity was not observed at any of the concentrations tested. Similarly, the MAC value was not found to be affected at 2 and 5 mM MgCl_2_ (Fig. 2C) or CaCl_2_ (Fig. 2D), concentrations higher than the physiological plasma concentrations of both these divalent cations [26]. However, an increase in MAC values was observed at higher concentrations of both these divalent cations.

### Effect of pH and bile salts

Cryptdin-2 decreased the trophozoite count in a concentration dependent manner in presence of bile salts and exhibited no change in its amoebicidal activity against *E. histolytica*. No change in MAC value was exhibited between the pH ranges of 6.5 to 7.5 while an increase in MAC value to 8 mg/L was observed at pH 8. At pH values 5 and 5.5, the observed MAC values were 32 mg/Land16 mg/L respectively.

### Morphological alterations induced by cryptdin-2

**Figure 3:**
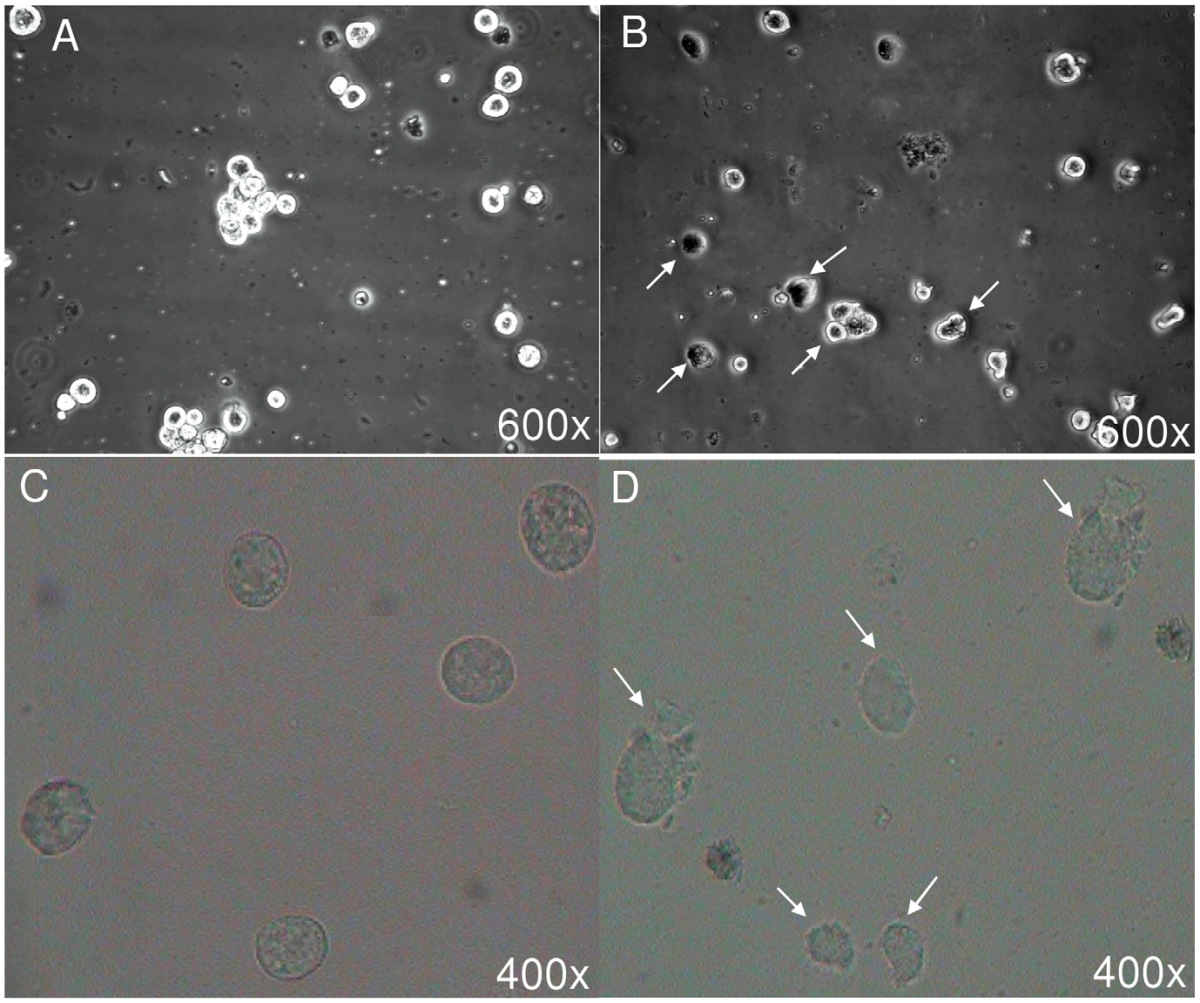
Morphological alterations induced by cryptdin-2 in *E. histolytica.* A) Phase contrast micrographs of normal untreated trophozoites of *E. histolytica* showing normal morphology without any alterations in structure and an intact plasma membrane (600×). B) Phase contrast micrographs of *E. histolytica* trophozoites treated with cryptdin-2 for an hour exhibiting altered morphology (arrows). The damage to the plasma membrane of the trophozoites is visible (arrows) (600×). C) Simple light photomicrographs of normal (untreated) trophozoites of *E. histolytica* as observed under simple light microscope at a magnification of 400×. D) Simple light photomicrographs of trophozoites of *E. histolytica* treated with cryptdin-2 for an hour as observed under simple light microscope at a magnification of 400×.The damage to plasma membrane is visible (arrows).

**Figure 4:**
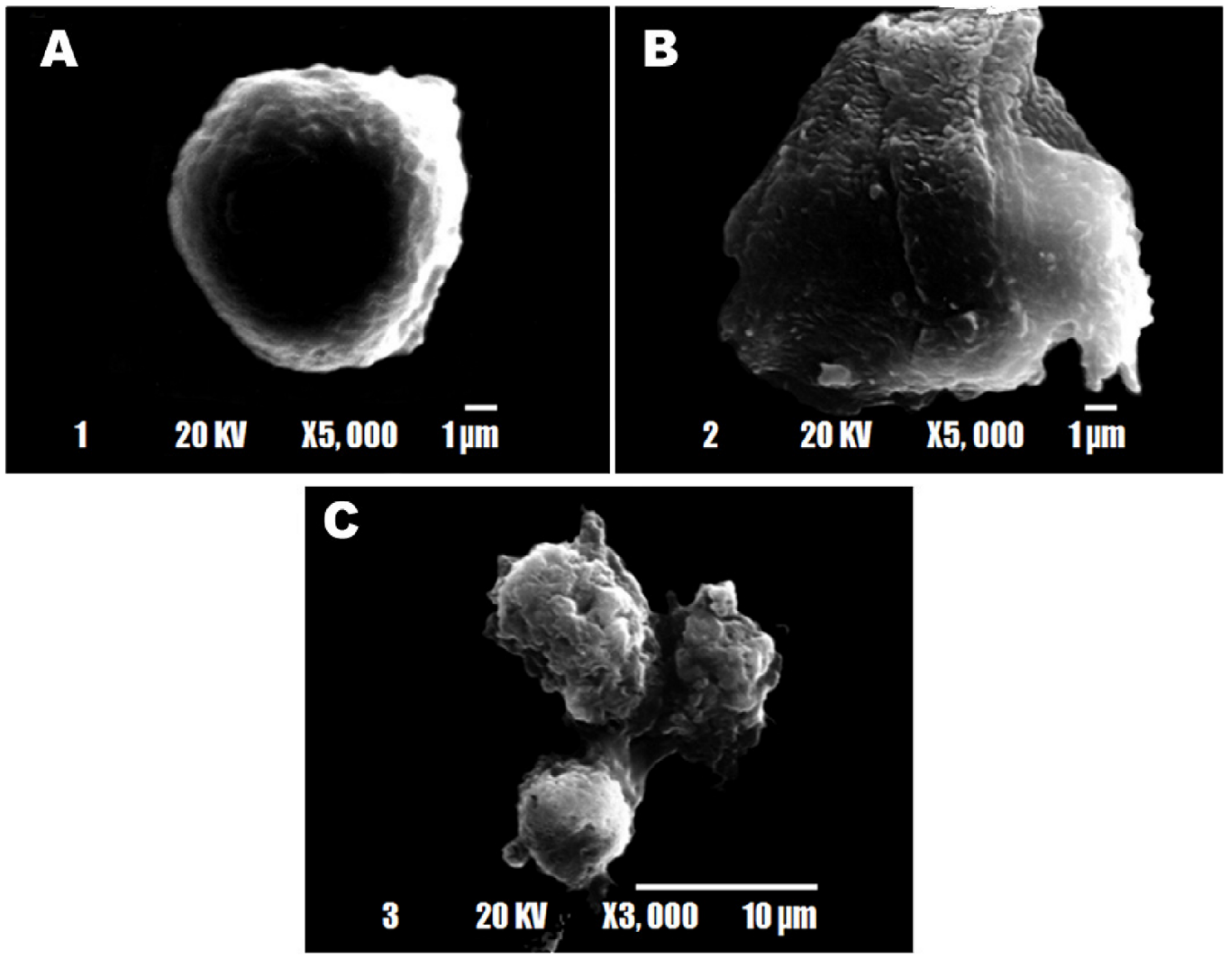
Scanning electron micrographs of cryptdin-2 treated *E. histolytica* cells. A) *E. histolytica* trophozoites showing normal morphology (5000×) B) Trophozoites showing the apparent leakage of cytoplasmic contents and the damaged plasma membrane after 60 minutes of treatment with cryptdin-2 (5000×). C) Trophozoites showing membrane wrinkling and abnormalities in surface morphology after incubation with cryptdin-2 for 60 min (3000×).

There was a marked change in the morphology of trophozoites treated with sub-inhibitory concentrations of the peptide with respect to controls which was quite evident from simple light (Fig. 3A–B), phase contrast (Fig. 3C–D) as well as scanning electron microscopic (Fig. 4A–C) studies. The deformation of trophozoites was clearly revealed by simple light as well as phase contrast microscopic techniques. Untreated *Entamoeba* trophozoites had smooth, normal surface morphology without any visible membrane abnormalities (Fig. 3A, 3C, 4A). It was indicated that cryptdin-2 could lead to complete disintegration of the cells after 1 h of treatment period and a majority of the cells appeared to have lost their membrane integrity (Fig. 3B, 3D, 4B, 4C). Scanning electron microscopic studies exhibited cell swelling and distortion of trophozoite morphology (Fig. 4B, 4C) and the damage to the plasma membrane was also apparent. It was interesting to note that in some of the cryptdin-2 treated trophozoites, the cytoplasmic components appeared to be bursting (Fig. 4B). The micrographs shown in Fig. 3 and Fig. 4 are representative of the ultra-structural damage of trophozoites, however, the damage was observed in each one of the fields analyzed.

### Membrane permeabilization assay

**Figure 5:**
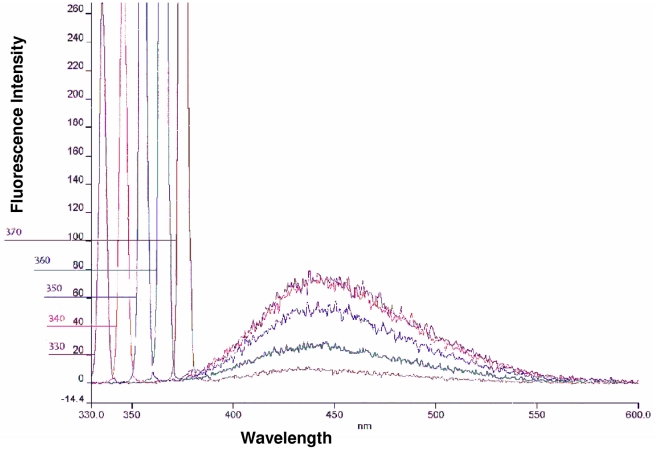
Fluorescence spectra of 1-*N*-phenylnaphthylamine (NPN). Fluorescence spectra obtained from a suspension of *E. histolytica* trophozoites in 5 mM/L HEPES buffer, pH 7·2 supplemented with 10 µM NPN. The measurement was done on a Perkin-Elmer luminescence spectrophotometer with a 5-nm excitation slit width.

**Figure 6:**
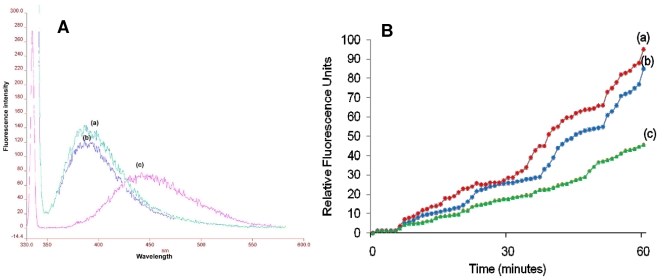
NPN uptake assay. A) Fluorescence spectrum of 10 µM 1-NPN excited at the wavelength of 340 nm (a) 1-NPN + cryptdin-2 (8 mg/L) (b) 1- NPN+EDTA, both at a concentration of 10 µM (c)) 1-NPN alone B) Increase in relative fluorescence units of10 µM 1-NPN at various time intervals a) 1-NPN+ cryptdin-2 (8 mg/L) (b) 1-NPN+ cryptdin-2 (4 mg/L) (c) 1-NPN+ cryptdin-2 (2 mg/L).

The series of emission spectra obtained with different excitation wavelengths (slit width, 5 nm) for NPN in presence of *E. histolytica* trophozoites exhibited an absorption maximum at approximately 415 nm. The most effective excitation wavelength was found to be 340 nm; an almost similar response was also obtained by exciting at 330 or 350 nm (Fig. 5). In the absence of *Entamoeba* trophozoites, NPN in HEPES buffer yielded a weak fluorescence peaking at 457 nm (excitation at 340 nm, data not shown). Incubation of the cells with NPN in presence of cryptdin-2 resulted in a marked blue shift in emission peak with increased magnitude of fluorescence intensity as compared to the intensity of the peak observed when the cells were incubated with NPN in absence of the peptide (Fig. 6A). Thus these results suggested that cryptdin-2 has the ability to permeabilize the membrane of *E. histolytica*. Moreover, relative fluorescence units (Fig. 6B) were also found to be significantly increased in a dose and concentration dependent manner in the presence of cryptdin-2 indicating the increased permeabilization of cryptdin-2 with time (as compared to controls).

### Effect on macromolecular synthesis

**Figure 7:**
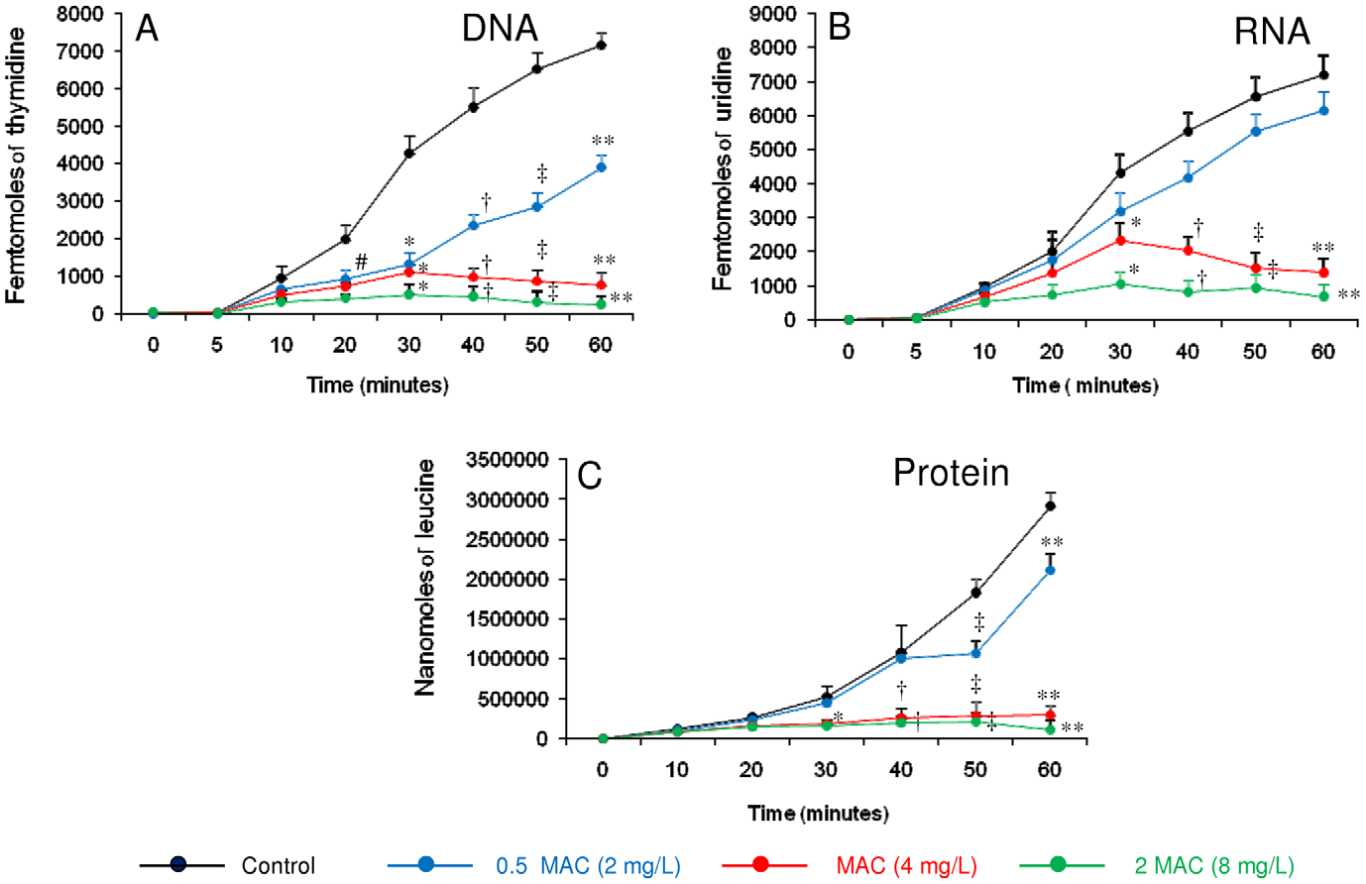
Effects of cryptdin-2 on macromolecular synthesis in *E. histolytica*. (A) [^3^H] - thymidine incorporation into DNA (B) [^3^H] uridine incorporation into RNA (C) L-[4, 5-^3^H (N)] leucine incorporation into protein were measured. The peptide was added at 0.5× MAC (2 mg/L), MAC (4 mg/L) and 2× MAC (8 mg/L). The results for control sample with no peptide are also shown. ^#^p < 0.05 vs DNA synthesis in control at 20 minutes, *p<0.05 vs DNA, RNA and protein synthesis in control at 30 min, † p<0.05 vs DNA, RNA and protein synthesis in control at 40 min, ‡ p<0.05 vs DNA, RNA and protein synthesis in control at 50 min, **p<0.05 vs DNA, RNA and protein synthesis in control at 60 min. Data representative of five separate experiments are shown.

To investigate whether cryptdin-2 affect macromolecular synthesis of *E. histolytica*, the incorporation of radioactive precursors viz [methyl-^3^H] thymidine, [5-^3^H] uridine and L-[4, 5-^3^H (N)] leucine into DNA, RNA and protein was studied in the presence of 0.5MAC, MAC and 2MAC of cryptdin-2. A dose and time dependent inhibition of DNA synthesis by cryptdin-2 was observed. However, after 60 minutes of exposure, DNA-synthesis was found to be increased in the control cells which were not exposed to the peptide. The percentage inhibition of incorporation of thymidine after 60 min was evaluated to be 45.69% (p<0.05), 89.34% (p<0.05) and 96.63% (p<0.05) in presence of 2 mg/L (0.5MAC) , 4 mg/L (MAC) and 8 mg/L (2MAC) respectively of cryptdin-2 (Fig. 7A). Similarly, the incorporation of RNA was also inhibited at the sublethal as well as higher concentrations of cryptdin-2. The percentage inhibition of uridine incorporation was 14.5%, 80.7% (p<0.05) and 90.43% (p<0.05)% in presence of 0.5MAC, MAC and 2MAC respectively of cryptdin-2 as compared to the control cells (Fig. 7B). Cryptdin-2 also exhibited a profound effect on protein synthesis by *Entamoeba histolytica* as the percentage inhibition of incorporation of leucine after 60 min was found to be 27% (p<0.05),89% (p<0.05) and 96% (p<0.05) in presence of 2 mg/L (0.5MAC), 4 mg/L (MAC) and 8 mg/L (2MAC) respectively of cryptdin-2 (Fig. 7C). Therefore, it can be concluded from these results that cryptdin-2 exerts the most significant effect on DNA synthesis followed by protein and RNA synthesis.

## Discussion

The lack of a useful alternative class of molecules against amoebiasis provides impetus to the efforts to identify and exploit alternative anti-amoebic therapies. Therefore, the present study evaluated the parasiticidal potential of cryptdin-2 against *E. histolytica*. Earlier, various cryptdin isoforms have been reported to exhibit parasiticidal activity against *Giardia lamblia* and it has been suggested that cryptdin-2 possesses the most potent giardicidal activity [20]. In the present study also, cryptdin-2 was able to inhibit the growth of *E. histolytica* in a concentration dependent manner. Interestingly, the amoebicidal concentrations of cryptdin-2 and metronidazole against *E. histolytica* were found to be at par in the current study. It can be inferred from this observation that cryptdin-2 can be exploited as an adjunct to metronidazole at lower concentrations against *Entamoeba* as has been reported recently against *Salmonella* [27]. The anti-amoebic activity (4 mg/L) observed in the present study seems to be comparatively higher than the giardicidal activity (20 mg/L) as reported previously [20]. The differing parasiticidal potency of cryptdin-2 against *Entamoeba* and *Giardia* can be attributed to the relative efficacy of binding to the trophozoite surface.

The peptide-target interactions are reported to be inhibited by divalent and to a lesser extent by monovalent cations. Therefore, in the current study, the stability of the peptide was also evaluated at approximate physiological concentrations of monovalent and divalent cations in colonic lumen [26], [28], [29]. MAC value was found to be increased to 32 mg/L in presence of 100 mM NaCl. Extracts from human intestinal biopsies containing AMPs have also been reported to exhibit diminished antimicrobial activity at 150 mM NaCl [30]. However, the amoebicidal activity was not affected at approximate physiological concentrations of bile salts, K^+^, Mg^2+^ and/or Ca^2+^ as well as at a broad pH range indicating its stability under *in-vivo* physiological conditions. Moreover, within the intestinal microenvironment (where the critical interaction of trophozoites and cryptdins occurs), the functional duality displayed by cryptdin-2 in terms of amoebicidal and immunomodulatory activity might be operative thereby combating the infection even in the presence of constantly differing concentrations of these salts [29].

To investigate the possible mechanism by which cryptdin-2 exerts its amoebicidal activity, morphology of peptide-treated trophozoites was examined. After 60 min of incubation with cryptdin-2, *E. histolytica* trophozoites revealed membrane wrinkling and probably leakage of cytoplasmic contents through the damaged cytoplasmic membrane. Although similar effects of other AMPs have been reported against various bacterial pathogens [31]–[34], this is the first report on cryptdin-2 induced morphological alterations in *Entamoeba histolytica* trophozoites. AMPs that disrupt membranes of pathogenic organisms are sometimes toxic to eukaryotic cells which questions their recommendation to be used as systemic drugs [35], [36]. Interestingly, cryptdin-2 exhibits very low cytotoxicity towards murine macrophages even at concentrations much higher than its effective microbicidal concentrations [19]. This difference in susceptibility has been attributed to the presence of cholesterol on eukaryotic cell membrane which stabilizes the lipid bilayers thereby protecting the eukaryotic cells from AMP-induced damage [37].

NPN permeabilization studies further evidenced this membrane-dependent mechanism of amoebicidal action of cryptdin-2. NPN fluoresces weakly in an aqueous environment but strongly in the hydrophobic interior of cell membranes. Upon destabilization of the cellular membrane by antimicrobial agents, the dye enters the damaged membrane, where it emits stronger fluorescence [38]. The marked blue shift accompanied by an increase in fluorescence intensity observed in the emission spectrum of NPN in presence of cryptdin-2 indicated the movement of NPN into a more hydrophobic environment. These observations were consistent with ultra structural findings indicating that cryptdin-2 was able to permeabilize the cytoplamsic membrane even at sub-lethal concentrations. Furthermore, a time and dose dependent increase in fluorescence was also observed in cells incubated with cryptdin-2 thereby indicating that the peptide treatment influenced membrane permeability. Therefore, both ultrastructural as well as fluorescence studies provided evidence that the surface of *E. histolytica* trophozoites was being modified by cryptdin-2 in order to exert it amoebicidal action. This finding confirms the earlier reports that Paneth cell cryptdins are natural pore forming peptides and may also be capable of mediating the transport of various therapeutic molecules inside the target cells [39].

In addition to membrane disruption, many studies have focused on intracellular effects through which AMPs bring about cell death. The cytoplasm contains an abundance of polyanionic molecules, such as nucleic acids and proteins, which may be the possible interaction sites for the cationic AMPs. In the present study, DNA, RNA and protein synthesis of *E. histolytica* was inhibited by cryptdin-2 in a time and dose dependent manner. It was also revealed that cryptdin-2 was more effective in inhibiting the incorporation of thymidine followed by leucine and uridine suggesting that DNA synthesis is more sensitive to its amoebicidal action. It is possible that membrane permeabilization affects the macromolecular synthesis due to leakage of cell contents and essential ions which are required for the activity of intracellular enzymes thereby interfering with essential metabolic processes inside the target cells [40]. Earlier also, inhibition of macromolecular synthesis has been reported for various AMPs like bactenectins [41], human neutrophil peptide-1 [42], pleurocidin [43] derived peptides and the epididymal defensin DEFB118 [44].

In conclusion, we report that cryptdin-2 exerts amoebicidal activity by inducing striking morphological changes in *E. histolytica* which is consistent with its membrane dependent mechanism of action. In addition to membrane permeabilization, its amoebicidal mechanism involves inhibition of DNA, RNA and protein synthesis. Given the antibacterial [19] as well as antiprotozoal efficacy of cryptdin-2, this peptide may be exploited as a broad spectrum antimicrobial agent. It may also be inferred that cryptdin-2, if not alone, may at-least be used in conjunction with metronidazole and/or other available anti-amoebic drugs in near future.
